# Pericardial sarcoidosis presenting as PUO diagnosed on FDG PET CT scan

**DOI:** 10.22038/aojnmb.2019.38132.1255

**Published:** 2020

**Authors:** Solav Shrikant Vasantrao, Patil Abhijit Mahaveer, Savale Shailendra Vasant, Salunke Deepak Vishnu

**Affiliations:** 1 Consultant, SPECT Lab, Nuclear Medicine Services, Pune, Maharashtra, India; 2 Director Niramaya Hospital, Chinchwad, Pune, Maharashtra, India

**Keywords:** PUO, Sarcoidosis, Pericardial, FDG PET CT

## Abstract

Pyrexia of unknown origin (PUO) is a common problem in day-to-day practice. FDG PET CT is an established investigation that aids in identifying the cause of PUO. Due to its high sensitivity PET detects an occult hypermetabolic focus in the body where CT helps in anatomical localization, vascularity, enhancement characteristics of the lesion detected on PET. It helps to differentiate benign versus malignant cause and target biopsy. Tuberculosis, lymphoma, pyelonephritis, thyroiditis appear hypermetabolic on FDG PET CT. Pericardial sarcoidosis is rare and not reported in literature as a cause of PUO. Presented here is a case of PUO secondary to pericardial granulomatosis diagnosed on PET CT. Cardiac MRI also helps in better tissue characterization and associated myocardial involvement of sarcoidosis. Histology confirmed the diagnosis of pericardial sarcoidosis in this case.

## Introduction

 With reducing cost, PET CT is being increasingly used in cases of fever of unknown origin. Common causes of PUO are bacterial infection as tuberculosis, hidden malignancies as lymphoma, autoimmune diseases as thyroiditis etc. High sensitivity of FDG PET enables early detection of lesions before morphologic changes set in. Other conventional imaging methods largely give anatomical information and depend upon manifestation of morphologic changes. FDG-PET CT is performed as a whole body procedure hence detects number and site of lesions not suspected clinically. We report a case of pericardial sarcoidosis suspected on PET CT and confirmed on histology.

## Case report

 A 44 years old male presented with 4 weeks of fever, breathlessness. There was no weight loss (90 kg). Physical examination showed tachycardia 125 beats per minute, tachypnoea (36/minute), normal blood pressure (110/80 mmHg). Soft systolic murmur was heard in left parasternal space. 

**Figure1:**
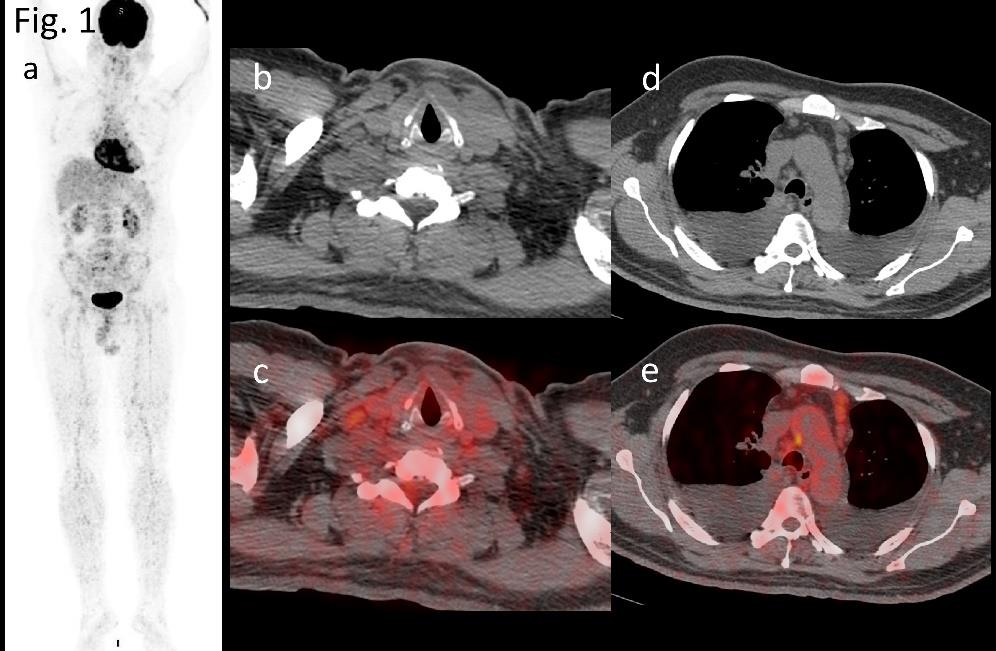
a) 3D MIP of whole body PET CT, b,d) Axial CT and c,e) hypermetabolic right supraclavicular and mediastinal (right paratracheal, pretracheal and left prevascular) nodes

**Figure 2:**
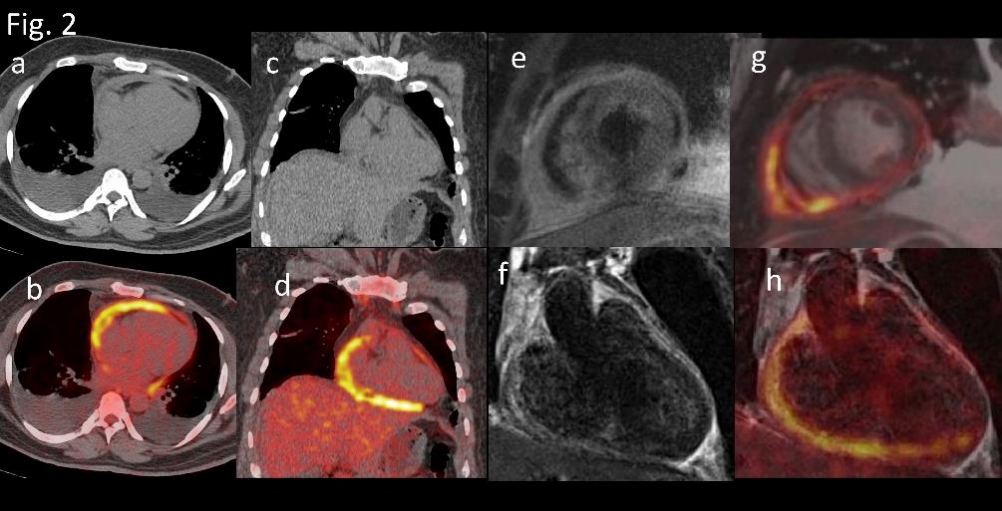
a,c,) Plain CT b,d,) PET CT images reveal hypermetabolic pericardial wall thickening and bilateral pleural effusion. e) Two chamber short and f) long axis T2TSE MRI and g,h) corresponding sequential fusion PET MRI reveal pericardial thickening appearing heterogeneously hyperintense on T2 WI corresponding to the hypermetabolic pericardial thickening on PET CT

 There was no obvious pericardial rub. Lungs had few rales. Abdomen was soft with no organomegaly. Hemoglobin was 11.9 gm/dl (range 12–16 gm/dl), WBC 7800/ µl (6000-10000/µl); platelets 414000/ µl (150000-450000/ µl); LDH (lactate dehydrogenase) 200 U/L (100-250); Blood Widal test excluded enteric fever. Sputum for AFB (acid fast bacilli) was negative for tuberculosis. Sonography showed bilateral pleural effusions, small pericardial effusion. There was no evidence of deep vein thrombosis on color doppler scan. FDG PET CT was performed using 7.7 mCi of ^18^F- fluorodeoxyglucose on 6 hours empty stomach. Scanning was done at 60 minutes using Siemens Horizon 16 slice PET CT system. The pericardium showed intense uptake of FDG in the anterior, inferior and right lateral walls. The anterior wall showed FDG avid thickening measuring 10×81mms standardized uptake value (SUV) 7.74. The inferior wall of pericardium showed thickening of 107×13mms with SUV value of 12.07. Few mediastinal lymph nodes were noted as follows: subcarinal node 17×13 mms SUV 3.86, left internal mammary node 17×6 mms SUV 2.58, right internal mammary node 8 mms SUV 2.81, left paratracheal 10 mms SUV 1.80, right paratracheal 10 mms SUV 3.24. Left supraclavicular node 19 mms SUV 2.53. Right level IV neck node 16 mms SUV 2.26 (Figure 1). Bilateral moderate pleural effusions and small ascites were noted. The myocardium did not show focal increased FDG uptake (Figure2a, b, c, d). Cardiac MRI was performed using T2 spin echo and TRUFI sequence on 1.5T Siemens Sempra MRI system. Sequential fusion of PET and MRI data was done on  station. MRI revealed diffuse asymmetric pericardial thickening hyperintense on T2W corresponding to PET CT (Figure 2e, f, g, h).

**Figure 3:**
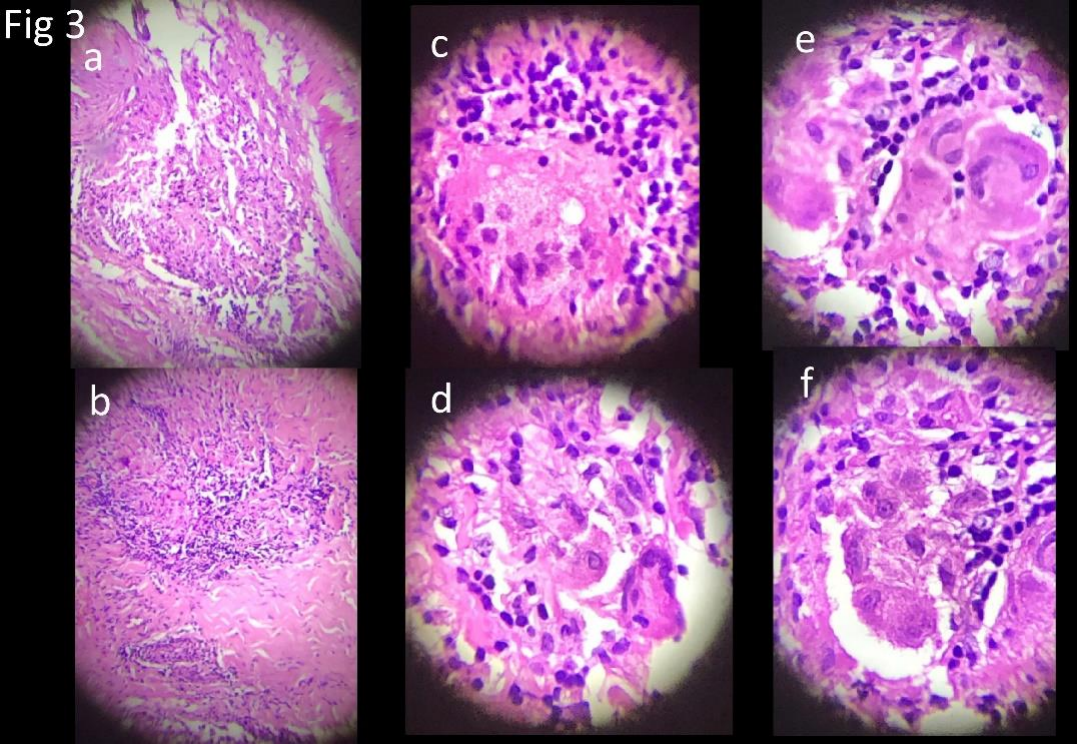
Microphotograph showing noncaseating epithelioid granuloma with multinucleate Langhans giant cell in different magnifications. There are areas of necrosis and surrounding lymphocytic infiltrate with sclerosis consistent with sarcoidosis

 In view of these findings a diagnosis of granulomatous disease involving the pericardium was made. Serum ACE (angiotensin converting enzyme) was recommended. The value was 72 U/L (normal 50). Tuberculin test was negative. Histology (pericardial window) showed non- caseating Granulomas, multinucleated Langhan’s giant cells and lymphocytic infiltrates (Figure 3).

 Steroids and empirical antitubercular treatment were initiated. Myocardial biopsy was not performed as FDG PET CT of myocardium was normal.

## Discussion

 The term sarcoidosis was introduced in 1899 by Caesar Boeck to describe skin lesions caused by epithelioid cells with pale nuclei and few giant cells. Due to its resemblance to sarcoma, he called these benign sarcoid of skin (1). The precise cause of sarcoidosis is unknown however, environ-mental exposure to insecticides, inorganic particles have been postulated (2). 

Propionibacterial and mycobacterial DNA and RNA have been identified using PCR technique. Antibodies to mycobacterium tuberculosis have been detected in serum samples of patients with sarcoidosis (3).

 Sarcoidosis may affect any organ in the body. Involvement of lungs may cause dyspnea, cough, wheezing, chest pain. Uveitis may cause blurring of vision or blindness. Eye pain, photophobia, redness may also occur secondary to eye involvement. Neuro-sarcoidosis may present as cranial nerve palsy, headache, ataxia, cognitive dysfunction and seizures. Parotid gland enlargement in sarcoidosis is also known as Mikulicz syndrome. Musculoskeletal involvement includes sarcoid arthropathy, Lofgren syndrome (erythema nodosum, hilar lymphadenopathy, polyarthralgia / polyarthritis). Heerfordt’s syndrome (fever- parotid enlargement-facial palsy- anterior uveitis). Fever, weakness, weight loss may be general symptoms. Hypercalcemia and/or hypercalciuria is found in considerable number of newly diagnosed patients of sarcoidosis (4). Pulmonary alveolar macrophage are thought to be the synthetic source of serum assayable1, 25-(OH) 2-D in sarcoidosis and one of the causes of altered calcium metabolism (5). 

 Myocardial sarcoid may present with acute left ventricular failure, tachyarrhythmias, conduction disturbances, valvular insufficiency or sudden death (6). Rarely pericardium is involved (7, 8). Pericardial sarcoidosis may or may not be associated with myocardial sarcoidosis. Pericardial sarcoidosis may present with pericardial effusion, cardiac tamponade, chronic constrictive pericarditis or as asymptomatic pericardial effusion. Pericardial sarcoidosis without myocardial involvement has good prognosis (9). Myocardial involvement in pericardial sarcoidosis has adverse prognosis because of conduction disturbances, arrhythmias, myocardial dysfunction leading to heart failure (10).

 A study showed myocardial involvement in 25% of patients with sarcoidosis in the USA, and accounted for as many as 13–25% of deaths due to sarcoidosis (11). Involvement of the pericardium is uncommon even in the presence of significant myocardial infiltration. It is observed in fewer than 10% of patients with cardiac sarcoidosis, and these patients usually remain asymptomatic. Echocardiography showed small pericardial effusions in 19% of patients with sarcoidosis (12).

Left ventricular free wall, septum and conducting system are involved in that order of frequency. Pericardial involvement results in restrictive pericarditis. It may rarely cause constrictive pericarditis (13).

 Negative Tuberculin test aids in distinguishing sarcoidosis from tuberculosis. Allergy to tuberculin skin test was observed in 87% of cases of sarcoidosis. A positive test requires extensive searched for tubercular focus (14).

^67^Ga citrate is an age-old radioisotope that localizes to the transferrin receptors of iron and lactoferrin of macrophages (15). As sarcoid granulomas are rich in macrophages the lesions appear to be “hot”. Panda (involvement of lacrimal glands- parotid and submandibular salivary glands) and lambda signs (mediastinal lymphadenopathy) have been described (16, 17). ^67^Ga citrate has been reported to localize in viral pericarditis, post aortocoronary bypass pericardial inflammation (18). There is significant diffuse cardiac uptake at 48 and 96 hours (19).

Other conditions with ^67^Ga-citrate uptake are lymphoma, amyloidosis, atrial thrombus, myocarditis, systemic sclerosis (20).

 FDG PET CT is a sensitive method to detect foci of inflammation. Sarcoid granulomas express glut-1 receptors. Consequently, there is increased FDG uptake (21). FDG PET is more sensitive than Ga-67 and carries three times less radiation (22). However, pericardial localization of FDG is nonspecific. It can be seen in tuberculosis, amyloidosis, viral pericarditis, radiation induced pericarditis, connective tissue disorder (23).

 PET has the advantage of imaging the whole body distribution of disease, which is difficult with MRI.

 Plain and dynamic contrast MRI provides non-invasive assessment of the heart for evaluation of cardiac sarcoidosis. MRI gives information about scar, oedema, perfusion defects and abnormal biventricular function. Late Gadolinium enhancement along the ventricular walls is the typical finding of myocardial sarcoidosis. MRI without PET risks failure to diagnose active myocardial inflammation seen early in the disease until it results in fibrotic changes which occur late. T2-weighted MRI shows inflammation of pericardium in form of edema but is less sensitive than PET, and also inflammation in areas of fibrosis is not easily detected (24).

 MRI has the advantage of no ionizing radiation, but it is contraindicated in patients with implanted pacemakers or defibrillators unless they are MRI compatible. Also care is required in the use of gadolinium contrast in patients with impaired kidney function. Pericardial involvement is seen as irregular and patchy pericardial thickening with enhancement on contrast MRI.

 There is a case report in a Japanese journal of pericardial tuberculosis presenting with fever (25).Pericardial sarcoidosis has been reported previously presenting with acute resistant pericarditis (26).

 Corticosteroids is the mainstay of therapy for patients with cardiac sarcoidosis as it prevents left ventricular remodelling when myocardium is involved and improves survival (26). Pericardiectomy is considered to be the treatment of choice for most patients with constrictive pericarditis and is it is associated with improved long-term survival rates (28, 29).

## Conclusion

 Primary pericardial sarcoidosis is a rare entity. PET scan aids in detection of pericardial inflammation to target biopsy. It also helps to detect inflamed lymph nodes that could be alternate site for biopsy to arrive at a diagnosis. Our case of pericardial sarcoidosis presented with PUO. Despite a diligent search, we were unable to find a report on pericardial sarcoidosis presenting with PUO.
